# A Lightweight Trust Mechanism with Attack Detection for IoT

**DOI:** 10.3390/e25081198

**Published:** 2023-08-11

**Authors:** Xujie Zhou, Jinchuan Tang, Shuping Dang, Gaojie Chen

**Affiliations:** 1 State Key Laboratory of Public Big Data, College of Computer Science and Technology, Guizhou University, Guiyang 550025, China; xujiezhou@ieee.org; 2 Department of Electrical and Electronic Engineering, University of Bristol, Bristol BS8 1QU, UK; shuping.dang@bristol.ac.uk; 3 5G/6G Innovation Center, Institute for Communication Systems, University of Surrey, Guidford GU2 7XH, UK; gaojie.chen@surrey.ac.uk

**Keywords:** trust mechanism, Internet of Things, trust attack, attack detection

## Abstract

In this paper, we propose a lightweight and adaptable trust mechanism for the issue of trust evaluation among Internet of Things devices, considering challenges such as limited device resources and trust attacks. Firstly, we propose a trust evaluation approach based on Bayesian statistics and Jøsang’s belief model to quantify a device’s trustworthiness, where evaluators can freely initialize and update trust data with feedback from multiple sources, avoiding the bias of a single message source. It balances the accuracy of estimations and algorithm complexity. Secondly, considering that a trust estimation should reflect a device’s latest status, we propose a forgetting algorithm to ensure that trust estimations can sensitively perceive changes in device status. Compared with conventional methods, it can automatically set its parameters to gain good performance. Finally, to prevent trust attacks from misleading evaluators, we propose a tango algorithm to curb trust attacks and a hypothesis testing-based trust attack detection mechanism. We corroborate the proposed trust mechanism’s performance with simulation, whose results indicate that even if challenged by many colluding attackers that can exploit different trust attacks in combination, it can produce relatively accurate trust estimations, gradually exclude attackers, and quickly restore trust estimations for normal devices.

## 1. Introduction

The Internet of Things (IoT) is a network framework merging the physical domain and the virtual domain through the Internet [1]. IoT devices can collect information, process data, and interact with other connected members automatically. Security issues are major concerns persistent throughout the development of IoT. In IoT paradigms requiring device cooperation, a device may not have the capacity and integrity to complete most assignments and behave in the interest of most participants. Trust management is responsible for building and maintaining a profile of a device’s trustworthiness in a network to ensure that most devices are trustworthy. It is crucial for applications depending on the collaboration among IoT devices to guarantee user experience [2]. In this section, readers can interpret trust as a particular level of the subjective probability with which an agent assesses that another agent or group of agents will perform a particular action [3]. Trust mechanisms for issues in the traditional security field may not acclimatize well to IoT applications due to the following technical characteristics of IoT [4]:

- Popular IoT paradigms are heterogeneous, where devices have varying capabilities and communicate with various protocols. As a result, it is challenging to create a trust mechanism that can apply to different applications via easy adaptation.
- IoT devices usually possess limited computing power and memory. A practical trust mechanism must balance the accuracy of trust estimations and algorithm complexity.
- IoT devices are numerous and ubiquitous. A trust mechanism should be scalable to remain efficient when the number of devices grows in a network.
- Mobile IoT devices such as smartphones and intelligent vehicles are dynamic, frequently joining and leaving networks. It complicates maintaining their profiles of trustworthiness for trust mechanisms.

Apart from these challenges, malicious devices can mislead evaluators into producing incorrect trust estimations by launching trust attacks. It should be a consideration for trust mechanisms [5]. Roles played by contemporary devices are more and more complex. The social IoT is a paradigm of this trend, where researchers introduce the concept of human social networks to study relations automatically built among devices whose roles can shift between the requester and server [6]. It may render trust attacks more attainable and profitable. For example, malicious attackers collude to exaggerate conspirators and slander other devices, aiming at the monopoly of service providers in a network. On the other hand, it facilitates communication among devices with different views, which is very helpful in locating trust attackers.

Researchers have proposed various trust mechanisms for different IoT networks, most of which adopt distributed architectures due to the quantity and ubiquity of IoT devices. Usually, a trust mechanism ultimately quantifies a device’s trustworthiness with a trust estimation derived from a data fusion process. Data fusion is responsible for utilizing a batch of descriptions or metrics of a device’s behavior with different times and sources to evaluate this device’s trustworthiness, as the core function of the trust mechanism. Bayesian inference or Dempster–Shafer theory [7] are widely used approaches for data fusion, applicable to different networks such as sensor networks and ad hoc networks [8,9,10,11,12,13]. Supported by related statistics principles, the former can accomplish data fusion through simple computing. Analogous to human reasoning, the latter permits expressing the extent of uncertainty related to an event rather than entirely recognizing or denying its authenticity. This property is useful when acquired information about an event is temporarily scarce. They can work in conjunction: the former processes data gathered from direct observation and the latter processes data provided by other devices [8,10]. For similar reasons to why Dempster–Shafer theory is adopted, there is research choosing fuzzy logic [14] or cloud theory [15] for data fusion. It is also common to construct experience-based formulas for data fusion to let a trust mechanism designed for a particular application fully consider the characteristics peculiar to this application [16,17,18,19,20]. For example, Chen et al. propose a trust mechanism for social IoT systems where data fusion produces three universal metrics related to the properties of a device’s performance in honesty, cooperativeness, and community interest. Further, they consist of an application-specific formula to compute a trust estimation [16].

However, the above summarized technical characteristics of IoT bring the following common challenges that remain to be addressed for many existing trust mechanisms, regardless of whether their data fusion employs theoretical or empirical foundations. Firstly, A trust mechanism designed to solve specific trust problems of several applications is hard to suit other applications via adaptation, although it is feasible to propose a universal trust mechanism [10]. Secondly, trust mechanisms employing distributed architectures and asking devices to share trust data cannot efficiently manage many devices due to their limited storage and communication capabilities. Thirdly, trust mechanisms often assume that devices can guarantee service quality, which does not apply to applications having inherent uncertainty. For example, interactions may occasionally fail due to noise interference in communication channels [15].

Moreover, the explanation of how parameters related to data fusion determine trust estimations is not detailed in many existing trust mechanism, leading to the undesirable dependency on operational experience with trial and error in the deployment phase. This problem is not unique to experience-based data fusion; it can also occur in theory-based data fusion. A trust mechanism with theory-based data fusion may require extra parameters beyond the underlying theories to provide special features. For example, to endow newer information more weight in data fusion, the trust mechanisms using Bayesian inference proposed in [8,10] utilize an aging factor and a penalty factor, respectively. Bayesian inference alone cannot provide this feature, where evidence collected in different periods is equivalent to being used to update the prior distribution. A poorly explicable parameter may limit a trust mechanism’s performance. For example, in [16], the presented simulation indicates that the variation in the proposed trust mechanism’s estimations is not significant when altering the parameter related to external recommendations. Some research proposes cryptography-based reliable approaches that can protect the integrity and privacy of data for healthcare [21] and vehicle [22] applications. However, devices competent in quickly performing encryption and decryption operations, such as the cloud servers and intelligent vehicles in this research, are not generally deployed in the IoT field.

Finally, solutions to trust attacks are often absent in existing trust mechanisms. In this paper, trust attacks refer to the following attacks on service defined in [5]: on-off attacks (OOAs), bad-mouthing attacks (BMAs), ballot-stuffing attacks (BSAs), discrimination attacks (DAs), self-promoting (SP) attacks, value imbalance exploitation (VIE), Sybil attacks (SAs), and newcomer attacks (NCAs). Although the following research gives analyses of how their data fusion mitigates influences from trust attacks or methods to identify trust attackers, there are several vulnerabilities: It may be accurate to assume that attackers are bad service providers for faulty devices [8], but this assumption is no longer relevant for devices having more functions nowadays. The behavior of an attacker launching a DA may be different in front of two observers. Comparison-based methods against BMAs like the ones in [13,16,23] may cause them to misjudge each other. The fluctuation in trust estimations alone cannot serve as an indication for trust attacks [24] because they are not the only reason of this fluctuation. Moreover, there is a lack of discussion surrounding DAs, collusion among malicious devices, and the ability to launch multiple trust attacks. Table 1 lists protection against trust attacks explicitly stated in the related literature on trust mechanisms.

Fog computing [26] has been a popular technique for addressing IoT security issues [4]. In recent research employing fog computing to design trust mechanism [27,28,29], a fog node serving as a forwarder or analyzer receives data from and disseminates results to devices. There is research [23,24,25,30,31,32] aimed at building bidirectional trust between devices and fog nodes for the case where a device needs to choose a service provider from nearby fog nodes. A fog node can complete some challenging tasks for conventional techniques such as processing big data from numerous devices, managing mobile devices, and giving responses with low latency [33]. Although fog computing is a handy tool for researchers, it cannot directly solve the three summarized problems, as they remain in this research.

Additionally, most trust mechanisms proposed in the literature derive a device’s trust estimation from two sources using direct information gathered during interactions with this device and indirect information about this device provided by other devices. Ganeriwal et al. proposed a structure consisting of two modules: the watchdog module and the reputation system module. The former receives data from a sensor during each interaction and outputs a metric of the credibility of these data using an outlier detection technique. The latter takes these metrics and external evaluations of this sensor to output a metric of whether this sensor is faulty to deliver incorrect data [8]. This structure facilitates improvement in the trust mechanism’s adaptability: the watchdog module processes direct information, and the reputation system module processes indirect information. For example, in addition to outlier detection, the watchdog module can utilize a weighted summation-based method [28] or machine learning to generate a metric.

Given these considerations, we proposed a Bayesian trust mechanism (BTM), which emphasizes researching the reputation system module. BTM does not rely on any specific IoT technique and takes simple input to address the challenge of heterogeneity, which requires two common assumptions to evaluate devices and to identify trust attackers by listening to feedback whose sources are diversified: first, devices frequently communicate with each other, and second, normal devices are in the majority. These are our contributions in detail:

- This paper proposes a new trust estimation approach by adapting data structures and algorithms used in the beta reputation system (BRS). For e-commerce trust issues, BRS’s feedback integration feature combines Bayesian statistics and Jøsang’s belief model derived from Dempster–Shafer theory to let data fusion fully utilize feedback from different sources [34]. It enables the BRS to produce more accurate trust estimations defined from a probabilistic perspective to quantify an IoT device’s trustworthiness. In contrast to previous research utilizing the two techniques, the data fusion of BTM enables the following novel and practical features: trust estimations that are universal, accurate, and resilient to trust attacks; efficient detection against various trust attacks; an option to combine fog computing as an optimization technique to address the challenges of scalability and dynamic; and a probability theory-explicable parameter setting.
- Based on the above trust evaluation, this paper proposes an automatic forgetting algorithm that gives more weight to newer interaction results and feedback in the computing process of trust estimations. It ensures that an IoT device’s trust estimation reflects the device’s current status in time, retards OOAs, and expedites the elimination of adverse influences from trust attacks. In contrast to conventional forgetting algorithms, this algorithm can automatically adjust this weight to achieve good performance. These two contributions form the trust evaluation module of BTM, which is less restricted by the heterogeneity of IoT and balances the accuracy of trust estimations and algorithm complexity.
- This paper proposes a tango algorithm capable of curbing BMAs by improving the processing of feedback in BTM as a precaution. Based on the trust evaluation module and hypothesis testing, this paper designs a trust attack detection mechanism that can identify BMAs, BSAs, DAs, and VIE to deal with high-intensity trust attacks. These two form the trust attack handling module of BTM.
- This paper conducts a simulation to corroborate the performance of BTM, where it is simultaneously challenged by inherent uncertainty and considerable colluding attackers with composite attack capabilities composed of BMAs, BSAs, and DAs. The presented results indicate that BTM can ensure that evaluators generate relatively accurate trust estimations, gradually eliminate these attackers, and quickly restore the trust estimations of normal IoT devices. This performance is better than existing trust mechanisms.

For the convenience of notation reference during the reading of subsequent sections in this paper, Table 2 lists all the notations used in BTM. (This paper continues to use the notation method in [34]. When a superscript and a subscript appear simultaneously, the former indicates an evaluator, the latter indicates an evaluatee, and a second subscript indicates a position in a sequence. Sometimes, they are omitted for the sake of simplicity if there is no ambiguity).

## 2. Materials and Methods

In this section, we elaborate on how BTM functions in this sequence: its system model; its basic trust evaluation approach. Given two probabilistic definitions of trust and reputation, this approach lets the evaluator regard direct interaction results as evidence to perform Bayesian inference; its feedback integration mechanism, where Jøsang’s belief model enables the evaluator to utilize external evidence from other devices as feedback in Bayesian inference; its forgetting mechanism; and its trust attack-handling module.

### 2.1. System Model

**Figure 1:**
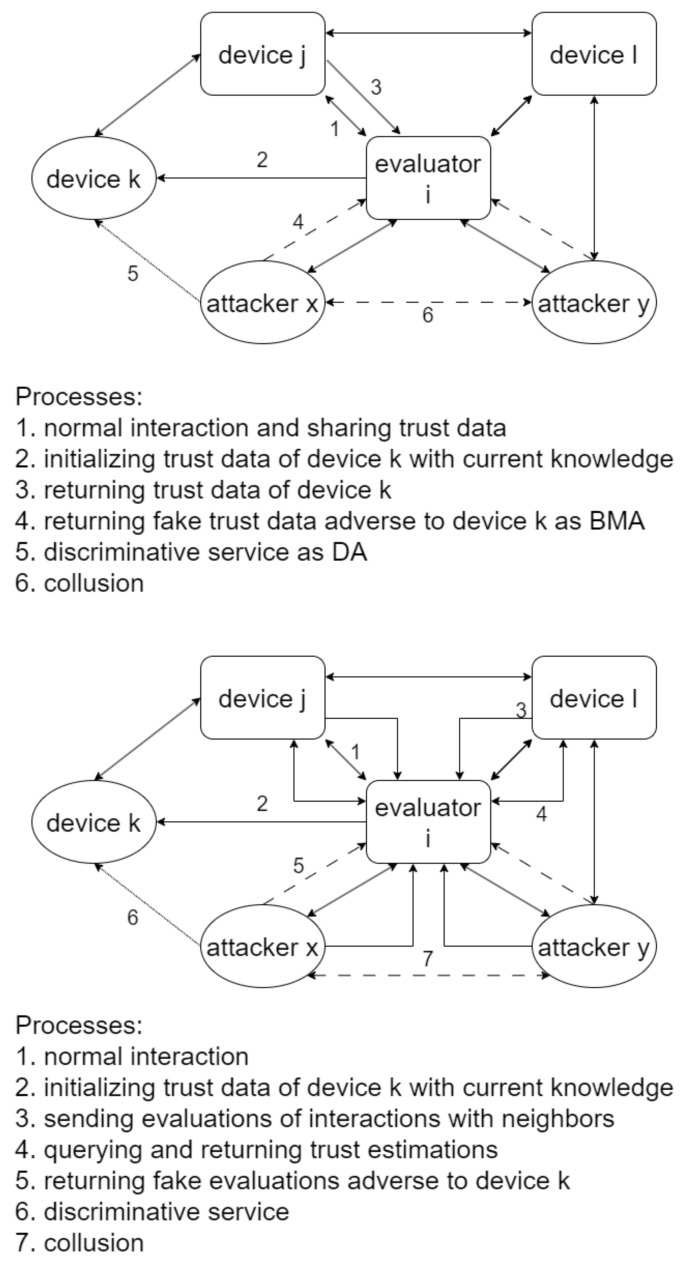
BTM’s architecture with two optional trust evaluation styles: purely distributed style and core style, illustrated from the view of evaluator *i*.

In BTM, devices are not necessarily homogeneous. The watchdog module generates a boolean value representing whether the device feels satisfied with the server’s behavior during an interaction. Each device determines the design of this module according to its specific requirements. The reputation system module takes input from the watchdog module and feedback from other devices to produce trust estimations, including all algorithms proposed in this paper. BTM offers two feedback integration algorithms, providing two optional trust evaluation styles for the same network. Their trust estimations are virtually identical given the same input. Figure 1 illustrates BTM’s architecture from the view of evaluator *i*.

In the purely distributed style, each device is equipped with the two modules and undertakes trust management on behalf of itself. Furthermore, devices need to share local trust data to ensure the accuracy of trust estimations. If a device has accomplished at least one interaction with device *i* lately, it is a neighbor of device *i*. When device *i* initiates contact with device *k*, it initializes the trust data of device *k* based on its current knowledge. Then, it requests the trust data of device *k* from its all neighbors to perform feedback integration. There are two colluding malicious devices *x* and *y* trying to mislead device *i* into producing trust estimations in more favor of them by trust attacks. As a neighbor of devices *i* and *k*, device *j* satisfies device *i*’s request. Meanwhile, the two attackers always return fake trust data adverse to device *k*. Attacker *x* also discriminates against device *k* by ignoring requests or providing trouble service. BTM should help device *i* solve the confusion why devices *k* and *x* criticize each other, as both them are good neighbors.

In the core style, a common device only has a watchdog module and directly submits its input as evaluations of other devices to a neighbor equipped with the two modules. It is responsible for the whole network’s trust management and disseminates trust estimations as the sole evaluator. This evaluator is elected by devices or designated by managers beforehand. The device can send an evaluation after an interaction or merge multiple evaluations into one before reporting to this evaluator. The evaluator periodically checks whether each device functions well by demanding service or self-check reports. This process is not necessary if it can receive adequate evaluations from neighbors that can guarantee their service quality because of a property of BTM’s feedback integration.

An application selects the better style according to its conditions. The main difference between the two styles is the scalability determinant: it hinges on the storage and communications abilities of most devices on average in the former, while it mainly depends on the storage and computing abilities of the sole evaluator in the latter. It is easier to strengthen this evaluator merely when wanting to accommodate more devices. Moreover, if devices merge evaluations and keep the frequency of interactions, the sole evaluator can invoke no more feedback integration algorithms than the purely distributed style. Given these considerations and that the research of elections among devices is not involved, we adopt the core style and assume that the sole evaluator is a fog node. The fog node is flexible to deploy and has more resources than devices to execute algorithms. Typically managed by a large organization such as a company, it is also more reliable [26].

BTM forbids a device from sending an evaluation of itself, which precludes SP attacks. BTM is expected to accurately and efficiently distinguish malicious devices that can use the following modeled trust attacks in combination:

- OOAs, attackers periodically suspend attacks to avoid being noticed;
- BMAs, attackers always send negative evaluations after interactions;
- BSAs, attackers always send positive evaluations after interactions;
- DAs, attackers treat other devices with a discriminatory attitude, providing victims with nothing or terrible service;
- VIE, attackers always send evaluations contrary to reality after interactions.

### 2.2. Trust Evaluation Based on Direct Observation

Since direct observation is the most fundamental approach to trust evaluation, our study starts with an abstraction of the definition of trust given in Section 1 from the perspective of probability theory to introduce Bayesian statistics to process results from direct interactions. In BTM, a trust value quantifies a device’s trustworthiness, derived from a reputation vector storing digested information from all previous interactions. Bayesian statistics enables initializing reputation vectors freely and updating them iteratively.

Given that device *j* accomplishes an assigned task with a probability $p_{j}$, we define device *i*’s trust in device *j* as its estimation of the probability of satisfying service from device *j* in the next interaction. It is desirable for device *i* that $t_{j}^{i}$ approximates $p_{j}$. In daily life, building trust by synthesizing what people have said and done in the past is deemed reasonable. In this kind of reputation-based trust model, reputation can be regarded as a random variable that is a function of previous behavior, and trust is a function of reputation. The two steps can be formulized as follows:

(1) $$\begin{array}{cc} {\mathrm{rep}}_{j}^{i} & =f_{1}\left(b_{j1}^{i},\cdots ,b_{jn}^{i}\right),\\ t_{j}^{i} & =f_{2}\left({\mathrm{rep}}_{j}^{i}\right), \end{array}$$

where $b_{jn}^{i}$ represents the behavior of device *j* observed by device *i* in the *n*th interaction, described by a random variable or a group of random variables. Updating the reputation given a new behavior is more convenient than updating the trust because the reputation can serve as a data structure containing digested information, and the trust’s form can be more intelligible for people.

Traditionally, a device can qualitatively describe the other side’s behavior in each interaction with a Boolean value. Such a value can serve as an instance of a random variable abiding by a binomial distribution B(1,θ), where θ represents an independent trial’s success rate unknown to this device. In Bayesian statistics, a device can refer to acquired subjective knowledge to estimate a parameter with a few samples called evidence and to update the result iteratively. For B(1,θ), θ’s prior distribution is a beta distribution:

(2) $$p\left(\theta ,\alpha ,\beta \right)=\frac{\theta^{\alpha -1}{\left(1-\theta \right)}^{\beta -1}}{\int \theta^{\alpha -1}{\left(1-\theta \right)}^{\beta -1}d\theta },$$

where α and β are hyperparameters set beforehand according to the domain knowledge of θ. The denominator is a beta function denoted by $\mathrm{Beta}\left(\alpha ,\beta \right)$. Note that the $\mathrm{Beta}\left(1,1\right)$ is identical to U(0,1), where θ is uniformly distributed over [0,1]. It is a reasonable prior distribution when the knowledge of θ is scarce. Given evidence, $data=\left(x_{1},x_{2},\ldots ,x_{n}\right)$ including *r* successful attempts and *s* unsuccessful attempts. The posterior distribution is obtained using Bayes’ theorem, characterized by a conditional probability density function:

(3) $$p\left(\theta \;|\;data,\alpha ,\beta \right)=\frac{\theta^{\alpha +r-1}{\left(1-\theta \right)}^{\beta +s-1}}{\mathrm{Beta}\left(\alpha +r,\beta +s\right)}.$$

Equation (3) is the prior distribution in the next estimation too. Rather than a posterior distribution giving all probabilities of an unknown parameter’s values, it is more common to output the expected value of this distribution in Bayesian parameter estimation. The expected value of (2) is $\frac{\alpha }{\alpha +\beta }$.

Given (1), BTM represents device *j*’s reputation at device *i* as ${\mathrm{rep}}_{j}^{i}=\left(\alpha_{j}^{i},\beta_{j}^{i},r_{j}^{i},s_{j}^{i}\right)$ and represents $t_{j}^{i}$ as $\frac{\alpha_{j}^{i}+r_{j}^{i}}{\alpha_{j}^{i}+\beta_{j}^{i}+r_{j}^{i}+s_{j}^{i}}$, where $r_{j}^{i}=\sum_{k=1}^{n}b_{jk}^{i}$ and $s_{j}^{i}=n-r_{j}^{i}$. Because a greater α or β brings about less variation in the trust when *r* or *s* changes, device *i* can increase $\alpha_{j}^{i}$ and $\beta_{j}^{i}$ if it has confidence in its knowledge of device *j*. As the evaluator should set α and β during the initialization of reputations, BTM does not suggest any operation on *r* and *s* without evidence-based reasons.

Note that the feedback integration and forgetting mechanisms introduced in the following content do not change the fact that a trust is a parameter estimation in nature, on which BTM relies to handle the heterogeneity and inherent uncertainty. The presented simulation will confirm that inherent uncertainty cannot mislead evaluators into misjudging normal devices even if meeting trust attacks. In the following content, trust values and reputation vectors in BTM are abbreviated to trusts and reputations.

### 2.3. Feedback Integration

Feedback integration enables updating reputations using external evidence contained in evaluations from other devices to expedite the acquisition of accurate trusts. It also retards DAs by synthesizing evaluations of a device from diversified views. Derived from the combination of Jøsang’s belief model with Bayesian statistics and formulized with group theory, BTM’s feedback integration can serve as a more accurate extension of BRS [34]. As illustrated in Section 2.1, BTM includes two feedback integration algorithms, providing two trust evaluation styles producing virtually identical trusts. The answer to which better hinges on applications. We also compare these algorithms with their counterpart in BRS. Note that BTM does not adopt the common practice that computes a global trust estimation by weight-summing direct and indirect trust estimations like [10,28]. In BTM, when an evaluator receives a piece of feedback, it directly digests this feedback’s influence on a device’s trust into this device’s reputation.

#### 2.3.1. Derivation of Feedback Integration

An evaluation’s effect should be proportional to the source’s trustworthiness, which is practicable by circulating the opinion defined in Jøsang’s belief model. Device *i*’s opinion about device *j* is $o_{j}^{i}=\left(b_{j}^{i},d_{j}^{i},u_{j}^{i}\right)$, where $b_{j}^{i},d_{j}^{i},u_{j}^{i}\in \left[0,1\right]$ and $b_{j}^{i}+d_{j}^{i}+u_{j}^{i}=1$. $b_{j}^{i}$ is the probability of a statement from device *j* being true in device *i*’s view, and $d_{j}^{i}$ is the probability of this statement being false. The sum of $b_{j}^{i}$ and $d_{j}^{i}$ is not bound to be in unity, and $u_{j}^{i}$ expresses device *i*’s uncertainty of this statement. In other words, they are belief, disbelief, and uncertainty. Device *j* sends $o_{k}^{j}$ as its evaluation of device *k* to device *i*. Device *i* processes $o_{k}^{j}$ using an operation called belief discounting [34] that

(4) $$\begin{array}{cc} o_{k}^{i:j} & =\left(b_{k}^{i:j},d_{k}^{i:j},u_{k}^{i:j}\right)=\left(b_{j}^{i}b_{k}^{j},b_{j}^{i}d_{k}^{j},d_{j}^{i}+u_{j}^{i}+b_{j}^{i}u_{k}^{j}\right). \end{array}$$

This process can be represented as a binary operation upon the opinion set $U_{o}$ that $o_{k}^{i:j}=o_{j}^{i}⊗o_{k}^{j}$. $\left(U_{o},⊗\right)$ is a monoid with an identity element (1,0,0).

On the other hand, the updating of reputations using evidence can be represented as a binary operation upon a subset of the reputation set $U_{r}=\left\{\left(c_{\alpha },c_{\beta },r,s\right)\;|\;r\geq 0,s\geq 0\right\}$, where α and β are constants. Given two reputations $a,b\in U_{r}$,

(5) $$a⊕b=\left(c_{\alpha },c_{\beta },a.r+b.r,a.s+b.s\right).$$

‘.’ denotes fetching a scalar in a vector. $\left(U_{r},⊕\right)$ is a commutative monoid. Its commutativity ensures no exception when simply adding positive and negative cases to merge evidence. In BTM, $o_{j}^{i}$ is determined by ${\mathrm{rep}}_{j}^{i}$ with a function from $U_{r}$ to $U_{o}$ defined in (6). It is a bijection, and the inverse function is (7). Algorithm 1 describes how device *i* integrates ${\mathrm{rep}}_{k}^{j}$ as an evaluation using these two equations. Equation (8) directly gives the result of the brief discounting. This algorithm precludes SP attacks because a sender cannot provide an evaluation of itself. Note that input parameters’ original values change when altering them in BTM’s algorithms.

(6) $$\begin{array}{c} o_{j}^{i}=g\left({\mathrm{rep}}_{j}^{i}\right)=\left(\frac{r_{j}^{i}}{\alpha_{j}^{i}+\beta_{j}^{i}+r_{j}^{i}+s_{j}^{i}},\frac{s_{j}^{i}}{\alpha_{j}^{i}+\beta_{j}^{i}+r_{j}^{i}+s_{j}^{i}},\frac{\alpha_{j}^{i}+\beta_{j}^{i}}{\alpha_{j}^{i}+\beta_{j}^{i}+r_{j}^{i}+s_{j}^{i}}\right). \end{array}$$

(7) $$\begin{array}{c} {\mathrm{rep}}_{j}^{i}=g^{-1}\left(o_{j}^{i},\alpha_{j}^{i},\beta_{j}^{i}\right)=\left(\alpha_{j}^{i},\beta_{j}^{i},\left(\alpha_{j}^{i}+\beta_{j}^{i}\right)\frac{b_{j}^{i}}{u_{j}^{i}},\left(\alpha_{j}^{i}+\beta_{j}^{i}\right)\frac{d_{j}^{i}}{u_{j}^{i}}\right). \end{array}$$

**Algorithm 1:** Feedback integration.**Input**: ${\mathrm{rep}}_{k}^{j}$, ${\mathrm{rep}}_{j}^{i}$, ${\mathrm{rep}}_{k}^{i}$**1** $o_{k}^{i:j}\leftarrow g\left({\mathrm{rep}}_{j}^{i}\right)⊗g\left({\mathrm{rep}}_{k}^{j}\right)$**2** ${\mathrm{rep}}_{k}^{i:j}\leftarrow g^{-1}\left(o_{k}^{i:j},\alpha_{k}^{i},\beta_{k}^{i}\right)$**3** ${\mathrm{rep}}_{k}^{i}\leftarrow {\mathrm{rep}}_{k}^{i}⊕{\mathrm{rep}}_{k}^{i:j}$

(8) $$\begin{array}{cc} & r_{k}^{i:j}=\frac{r_{k}^{j}\left(\alpha_{k}^{i}+\beta_{k}^{i}\right)r_{j}^{i}}{\left(s_{j}^{i}+\alpha_{j}^{i}+\beta_{j}^{i}\right)\left(\alpha_{k}^{j}+\beta_{k}^{j}+r_{k}^{j}+s_{k}^{j}\right)+r_{j}^{i}\left(\alpha_{k}^{j}+\beta_{k}^{j}\right)},\\ & s_{k}^{i:j}=\frac{s_{k}^{j}\left(\alpha_{k}^{i}+\beta_{k}^{i}\right)r_{j}^{i}}{\left(s_{j}^{i}+\alpha_{j}^{i}+\beta_{j}^{i}\right)\left(\alpha_{k}^{j}+\beta_{k}^{j}+r_{k}^{j}+s_{k}^{j}\right)+r_{j}^{i}\left(\alpha_{k}^{j}+\beta_{k}^{j}\right).} \end{array}$$

Note that $r_{k}^{j}$ suffers more discounting when the subjective parameters related to device *j* increase. Moreover, when $\alpha_{k}^{i}=\alpha_{k}^{j}$ and $\beta_{k}^{i}=\beta_{k}^{j}$ to let ${\mathrm{rep}}_{j}^{i}$ be comparable with ${\mathrm{rep}}_{k}^{i}$, $r_{j}^{i}=\infty$ is the only way to exempt $r_{k}^{j}$ from discounting:

$$r_{k}^{i:j}=\lim_{r_{j}^{i}\to \infty }\frac{\left(\alpha_{k}^{i}+\beta_{k}^{i}\right)r_{k}^{j}}{\frac{\left(s_{j}^{i}+\alpha_{j}^{i}+\beta_{j}^{i}\right)\left(\alpha_{k}^{j}+\beta_{k}^{j}+r_{k}^{j}+s_{k}^{j}\right)}{r_{j}^{i}}+\left(\alpha_{k}^{j}+\beta_{k}^{j}\right)}=r_{k}^{j}.$$

Algorithm 1 is suitable for the purely distributed style, where devices should periodically share their reputation data for the sake of feedback integration. Evaluator *i* prepares two reputations for device *j*: one only comprises evidence from interactions, while the other synthesizes both direct and discounted external evidence. The former is the base for the latter and is provided for other devices as the evaluation of device *j*. The latter is the base for $t_{j}^{i}$ and discounting evaluations from device *j*. Note that when evaluator *i* has integrated an old ${\mathrm{rep}}_{k}^{j}$, it needs to compute the latter reputation from scratch if it wants to update with a newer ${\mathrm{rep}}_{k}^{j}$.

#### 2.3.2. Incremental Feedback Integration

With the above practice, the device’s storage and communication abilities for saving and sharing reputations on average determine the max member number of BTM. Adapted from Algorithm 1 according to $f\left(x+\Delta x\right)\approx f\left(x\right)+f^{\prime }\left(x\right)\Delta x$, Algorithm 2 concentrates all trust management tasks in a network to a sole evaluator. Imposing minimal trust management burdens on common devices and not requiring sending an evaluation’s duplicates to different receivers, Algorithm 2 can extend BTM’s scalability simply by strengthening the sole evaluator. Moreover, it can update a reputation iteratively with new evaluations rather than from scratch and can endow the evaluator with a global view to estimate devices and to detect trust attacks. Algorithm 2 applies to applications where cooperative devices have differential performance, such as smart home applications managing smart appliances using a smartphone or wireless router. Even if composed of homogeneous or dynamic devices like intelligent vehicles, applications can also adopt Algorithm 2 with the help of fog nodes [14,33].

Δrep from the device substitutes rep as the evaluation in Algorithm 2, which is the increment of rep. That is, restricting α and β to be constants, evidence is gathered from recent interactions with a device and sent out since the last sending evaluation. α and β are fixed to unities in common devices because they are not deeply involved in the details of generating trusts anymore. Evaluator *i* cannot know $r_{k}^{j}$ and $s_{k}^{j}$ directly using Δrep. Therefore, they are saved in a vector where ${\mathrm{evi}}_{k}^{i:j}=\left(m_{k}^{i:j},n_{k}^{i:j}\right)=\sum_{l=1}^{n}\Delta {\mathrm{rep}}_{kl}^{j}$. The disc function discounts Δrep, where $\alpha_{k}^{j}+\beta_{k}^{j}$ is replaced by two. Note that $\Delta {\mathrm{rep}}_{k}^{j}$ is a direct observation result in device *j* while an evaluation needs to be discounted in fog node *i*. In BTM, $\Delta {\mathrm{rep}}_{k}^{j}$ is called direct evidence in the former case and feedback in the latter case.

Additionally, (9) is the equation for integrating positive feedback in BRS:

(9) $$r_{k}^{i:j}=\frac{{2r}_{k}^{j}r_{j}^{i}}{\left(2+\beta_{j}^{i}\right)\left(2+r_{k}^{j}+s_{k}^{j}\right)+{2r}_{j}^{i}}.$$

**Figure 2:**
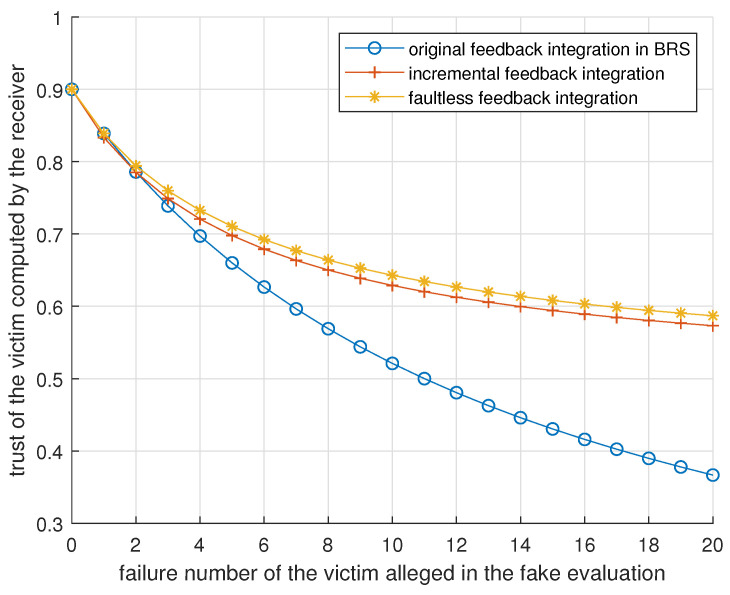
Trust of the victim per round in different feedback integration modes when consecutive criticisms are met.

To enable the free initialization of a device’s reputation even if without evidence, BTM separates α and β from *r* and *s* when representing this reputation and alters the mapping from reputations to opinions, resulting in the difference between (8) and (9). In the elementary form of providing feedback in BRS, the sender evaluates an agent’s performance in a transaction with a pair $\left(r,s\right)$ where r+s=w and *w* is a weight for normalization. The evaluator discounts this pair using (9) [34]. However, $r_{k}^{i:j}$ is a concave function of $r_{k}^{j}$. But, evaluator *i* cannot directly know the $r_{k}^{j}$ related to all previous transactions with this pair. The sender should add the pair of the new transaction to the pair of previous transactions and send this sum as the evaluation. Note that as the evaluation to be integrated in Algorithm 1, ${\mathrm{rep}}_{k}^{j}$ includes all evidence of previous interactions between devices *j* and *k*. This concavity provides some resistance against BMAs and BSAs, as Figure 2 shows, where ${\mathrm{rep}}_{j}^{i}=\left(1,1,8,0\right)$, ${\mathrm{rep}}_{k}^{i}=\left(1,1,8,0\right)$, and ${\mathrm{rep}}_{k}^{j}=\left(1,1,0,0\right)$ at the outset. $s_{k}^{j}$ increases by 1, and device *j* sends ${\mathrm{rep}}_{k}^{j}$ and $\Delta {\mathrm{rep}}_{k}^{j}$ to device *i* per round. The following sections assume that α=β=1 initially and that there is a fog node running Algorithm 2.
**Algorithm 2:** Incremental feedback integration.
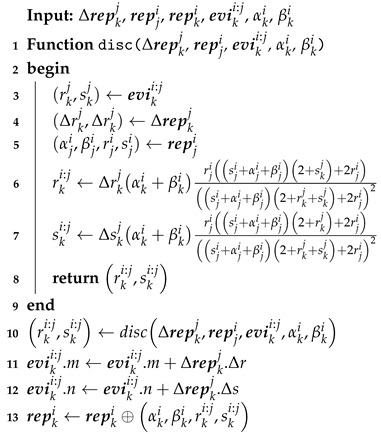


### 2.4. Forgetting Algorithm

In Section 2.3, integrating direct evidence and discounted feedback in any order will lead to the same reputation since $\left(U_{r},⊕\right)$ is commutative. However, a device does not necessarily behave smoothly; it may break down due to fatal defects or shift to the attack mode due to an OOA. A forgetting algorithm lets newer collected data carry more weight in trust evaluations to ensure that a device’s trust estimation reflects its latest status in time. If the target value $tar_{n}$ after the *n*th interaction is derived from a statistic of the *n*th interaction $stat_{n}$ and previous statistics, a common forgetting form like the one used in BRS [16,28,34] is

(10) $$tar_{n}=\lambda^{\left(n-1\right)}stat_{1}+\lambda^{\left(n-2\right)}stat_{2}+\cdots +\lambda^{0}stat_{n},$$

which uses a forgetting factor λ<1.

As the first example of utilizing the separated subjective and objective parameters in reputations, we proposed Algorithm 3, which can achieve the same forgetting by automatically adjusting these parameters. Its idea is that given α and β embodying subjective information related to the trust, evaluator *i* stores direct evidence of device *j* over a queue $q=\left(\Delta {\mathrm{rep}}_{0},\Delta {\mathrm{rep}}_{1},\cdots ,\Delta {\mathrm{rep}}_{n-1}\right)$ containing *n* evidence at most. The smaller the evidence’s subscript is, the older it is. The oldest evidence is then discarded and becomes the experience to update α and β when new evidence arrives in a full queue. Evaluator *i* also merges discounted feedback into the element at the queue’s rear. Using a single queue containing the two kinds of evidence can reduce the algorithm’s complexity and memory space with negligible deviations.

In Algorithm 3, $pop\left(q,x\right)$ lets the oldest element quit q and gives its value to x. q’s capacity ϕ varies with circumstances. A larger ϕ reduces the standard deviation of trusts but requires more memory space. The evaluator saves feedback in two double-dimensional arrays represented by two matrices *M* and *N*. M[j][k] denotes the element of the *j*th row *k*th column of *M*. Given the two matrices, ${\mathrm{evi}}_{k}^{i:j}.m=M^{i}\left[j\right]\left[k\right]$ and ${\mathrm{evi}}_{k}^{i:j}.n=N^{i}\left[j\right]\left[k\right]$, indexes and serial numbers start with zero. The for-loop updates elements where device *j* is an evaluation sender in $M^{i}$ and $N^{i}$ because $q_{j}^{i}$ brings $r_{j}^{i}$ an upper bound. Without this operation, the evaluation’s effect will indefinitely decline with the analysis of the concavity of (8) in Section 2.3. *v* is a random variable abiding by U(0,1), used to choose which matrix to update because the order of the arrival of feedback is not recorded.

As explained in Section 2.3, the evaluator prepares two kinds of reputations for a device in the purely distributed style. Therefore, The evaluator also maintains the two queues of these reputations and updates them simultaneously in Algorithm 3. Moreover, when the evaluator sends out a reputation as an evaluation, it can append the corresponding queue to this reputation. Then, the receiver merges this queue into its one. In this way, the sender and the receiver can forget the same evidence at about the same time.

This algorithm automatically sets the evidence’s weight: The initial values of α and β are $\alpha_{0}$ and $\beta_{0}$, $\alpha_{0}+\beta_{0}=c$. $\alpha_{1}=\frac{c\left(\alpha_{0}+\Delta r_{0}\right)}{c+\Delta r_{0}+\Delta s_{0}}$ when $\Delta {\mathrm{rep}}_{0}$ quits. Then, $\Delta {\mathrm{rep}}_{1}$ quits, resulting in $\alpha_{2}=\frac{c^{2}\left(\alpha_{0}+\Delta r_{0}\right)}{\left(c+\Delta r_{1}+\Delta s_{1}\right)\left(c+\Delta r_{0}+\Delta s_{0}\right)}+\frac{c\Delta r_{1}}{c+\Delta r_{1}+\Delta s_{1}}$. That is, the forgetting factors of the first and second rounds are $\frac{c}{c+\Delta r_{0}+\Delta s_{0}}$ and $\frac{c}{c+\Delta r_{1}+\Delta s_{1}}$, respectively. By mathematical induction,

(11) $$\begin{array}{cc} \alpha_{n} & =\frac{c^{n}\left(\alpha_{0}+\Delta r_{0}\right)}{\left(c+\Delta r_{n-1}+\Delta s_{n-1}\right)\cdots \left(c+\Delta r_{0}+\Delta s_{0}\right)}\\ & +\frac{c^{n-1}\Delta r_{1}}{\left(c+\Delta r_{n-2}+\Delta s_{n-2}\right)\cdots \left(c+\Delta r_{0}+\Delta s_{0}\right)}\\ & +\cdots +\frac{c\Delta r_{n-1}}{c+\Delta r_{n-1}+\Delta s_{n-1}}. \end{array}$$

**Algorithm 3:** Forgetting algorithm.

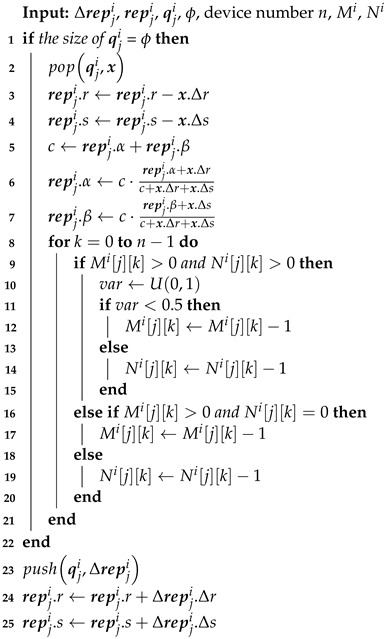



### 2.5. Module against Trust Attacks

Algorithms 2 and 3 cannot guarantee the accuracy of trusts in the face of trust attacks. In this section, we analyze the ability and limitation of BTM’s feedback integration against trust attacks first to clarify the aims of BTM’s trust attack handling module. This module consists of a tango algorithm that can curb BMAs by adapting Algorithm 2 and a hypothesis testing-based trust attack detection mechanism against BMAs, DAs, BSAs, and VIE.

#### 2.5.1. Influences of Trust Attacks and Tango Algorithm

For DAs, Algorithm 2 synthesizes feedback from different perspectives to render it unprofitable. For BMAs and BSAs, a reckless attacker sends a lot of fake feedback in a short time, which is inefficient due to the concavity of (8), illustrated in Figure 2. A patient attacker sends fake evaluations with an inconspicuous frequency for long-term interests, which works because Algorithm 2 does not check the authenticity of feedback.

Applying the principle that it takes two to tango to curb BMAs, Algorithm 4 is an adaptation of Algorithm 2. It divides blame between two sides when processing negative feedback, where the side having higher trust is given more Δs to criticize the other side. Assuming most interactions between normal device success, Algorithm 4 renders BMAs lose–lose with O(1) time-complexity extra computing to make an independent BMA attacker’s trust continuously decline. Algorithm 1 can be adapted with the same idea as Algorithm 4.
**Algorithm 4:** Tango algorithm.
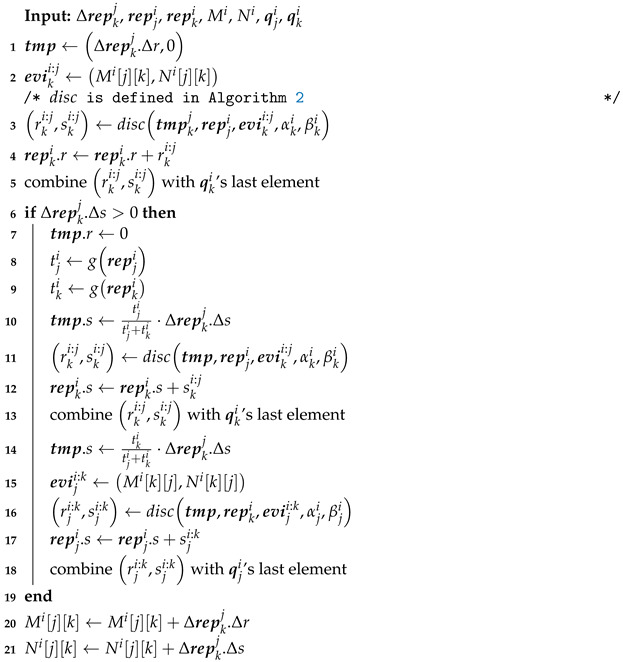


#### 2.5.2. Trust Attack Detection

Algorithm 4 mitigates trust attacks when normal devices are in the majority to tolerate some malicious devices. We propose a trust attack detection mechanism to identify attackers for harsher circumstances, which works in parallel with Algorithm 4 because the latter can filter subjects for the former.

BTM saves feedback in *M* and *N* for feedback integration. They also correspond to directed graphs in graph theory. If device *j* sends criticism of device *k*, there is an edge from node *j* to node *k* whose weight is N[j][k]. DAs and BMAs can cause abnormal in-degree and out-degree in *N*, respectively. BSAs can cause an abnormal out-degree in *M*. It is an outlier detection problem and has universal solutions [35]. For example, The local outlier factor (LOF) algorithm [36] can check the degrees of *n* nodes with $O(n^{2})$ time complexity.

BTM uses a new approach quicker than LOF to detect these anomalies. With BTM’s feedback integration, a device’s trust is a parameter estimation hard to manipulate using trust attacks. If $M^{i}\left[j\right]\left[k\right]=m$ and $N^{i}\left[j\right]\left[k\right]=n$, device *j* reports that device *k* succeeds *m* times and fails *n* times in recent interactions. Hypothesis testing can check its authenticity, whose idea is that a small probability event is unlikely to happen in a single trial. Using a *p*-value method, the null hypothesis is that device *j* honestly sends feedback, the test statistics are $M^{i}\left[j\right]\left[k\right]$ and $M^{i}\left[j\right]\left[k\right]$, the corresponding p-value denoted by ω is:

(12) ω=m+nmtkim1−tkin.

If the null hypothesis is true, ω should not be less than a significance level like 0.05 denoted by γ. In Algorithm 5, against BMAs, BSAs, and VIE, if $t_{j}^{i}<\zeta$, evaluator *i* calculates ω along the *j*th row. $\gamma_{1}$ is for patient attackers, which can tolerate a frequency of rejected null hypothesis no more than η. $\gamma_{2}$ is very tiny for reckless attackers. This algorithm can check a single node with O(n) time complexity. Note that Algorithm 3 makes m+n hover around ϕ.

The DA detection algorithm (Algorithm 6) is obtained by adapting Algorithm 5 via calculating ω along a column and deleting $\gamma_{2}$. Although the purely distributed style does not need *M* and *N* for feedback integration, it can introduce the two matrices to use Algorithm 5, whose updating is simple: when device *i* receives ${\mathrm{rep}}_{k}^{j}$ from device *j*, it changes the elements of the *j*th row *k*th column in $M^{i}$ and $N^{i}$ to ${\mathrm{rep}}_{k}^{j}$.
**Algorithm 5:** Detection against BMAs, BSAs, and VIE.
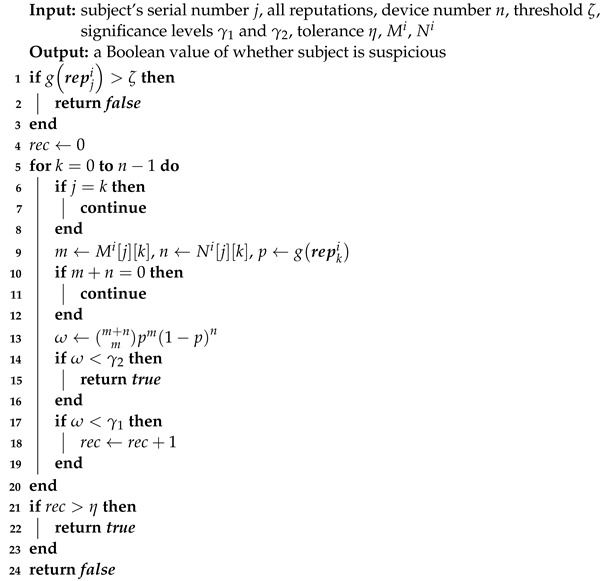


**Algorithm 6:** Detection against DAs.

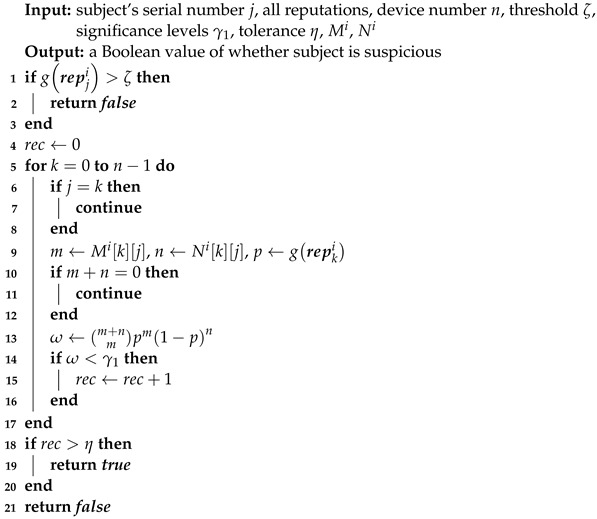



## 3. Results

In this section, we corroborate that even if challenged by high-intensity trust attacks, BTM can accurately estimate normal devices and identify malicious devices for applications having inherent uncertainty by simulation. Note that this paper adopts the core trust evaluation style and assumes that the sole evaluator is a fog node. The platform is a host computer with AMD Ryzen 5700G, 16 GB RAM, and Windows 11 home edition. The program is written in C++ and compiled using MSVC (19.29.30137 edition for ×64).

### 3.1. Design, Trust Attack Tactics, and Metrics

Devices and fog nodes are simulated using independent threads whose execution sequences, and the results are unpredictable. To simulate the inherent uncertainty caused by various adversary factors with different sources such as physical environments and networks, an interaction between two devices ends successfully with a probability of 0.8. Denoted by $n_{0}$, there are three initial device numbers: 10, 20, and 50. A device uniformly chooses a server from the other devices and sleeps for 1 millisecond after evaluating this interaction. The number of requests is limited to $20n_{0}$, representing a device’s lifespan. When a device expires, it becomes inaccessible, and the fog node archives its trust data. *n* denotes the number of active devices. The fog node also forces a suspicious device to expire in advance to remove it. The fog node periodically performs a series of operations: requesting service from each device, digesting received feedback, applying the two trust attack detection algorithms to each device, and adjusting the interval by which it can receive about $n^{2}$ feedback before the next round.

Attackers act normally first to build credible profiles within several requests when created, which is a simple OOA tactic. An independent attacker’s latency ranges within $\left[0,\frac{1}{4}\right]$ of $20n_{0}$. A colluding attacker’s latency is $5n_{0}$ to maximize the impact of attacks. There are three types of attackers. A fox is an independent BMA attacker sending a negative feedback after an interaction. A miser is a colluding DA attacker rejecting requests not coming from a conspirator or fog node. A hybrid is a stronger miser that can launch BMAs and BSAs. It sends positive feedback after an interaction when the server is a conspirator. Otherwise, it sends negative feedback. Table 3 lists the parameter values related to the simulation setting and BTM’s algorithms. The fog node judges a device as suspicious if its trust is below 0.5 or fails to pass the trust attack detection.

There has been no widely accepted benchmark comparing the performance of trust mechanisms due to the diversity of underlying theories for their design. For the aspect of identifying trust attackers, we borrow five metrics of classifiers in machine learning: precision is the proportion of true positives in all positives; recall is the proportion of true positives in all attackers; specificity is the proportion of true negatives in all normal devices; the accuracy is the proportion of true positives and true negatives in all devices; F1 score is the harmonic mean of precision and recall; average deviation is the average of the absolute value of the normal device’s final trust minus 0.8; average attacker trust is the average of the attacker’s final trust; and check count is the frequency of checking a device with a trust less than η.

The following presented data are averaged over 2000 repeated trials. Precision, recall, F1 score, and average attacker trust are not quite relevant when all devices are normal. The fog node records the average trusts of normal devices per 0.5 s when $n_{0}=50$.

### 3.2. Presentation

**Figure 3:**
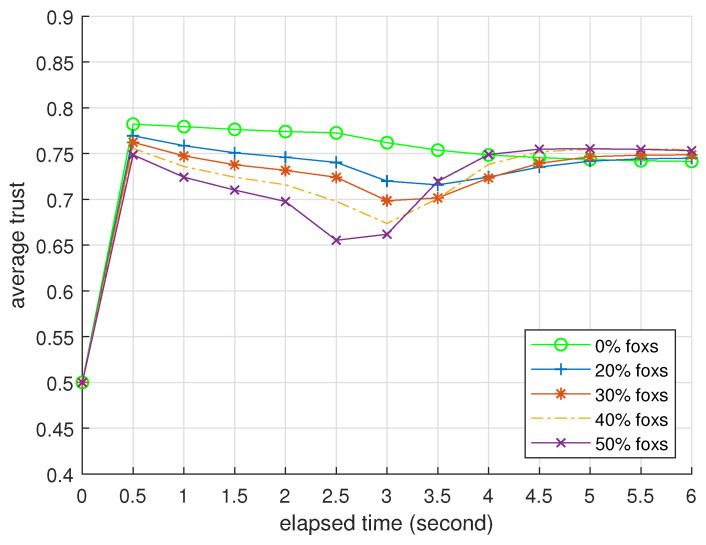
Average trusts of normal devices per 0.5 s, $n_{0}=50$; all attackers are foxes.

**Figure 4:**
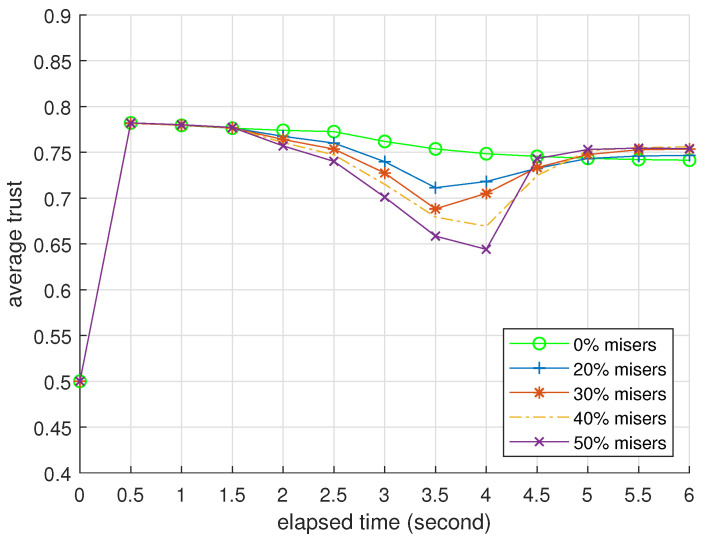
Average trusts of normal devices per 0.5 s, $n_{0}=50$; all attackers are misers.

**Figure 5:**
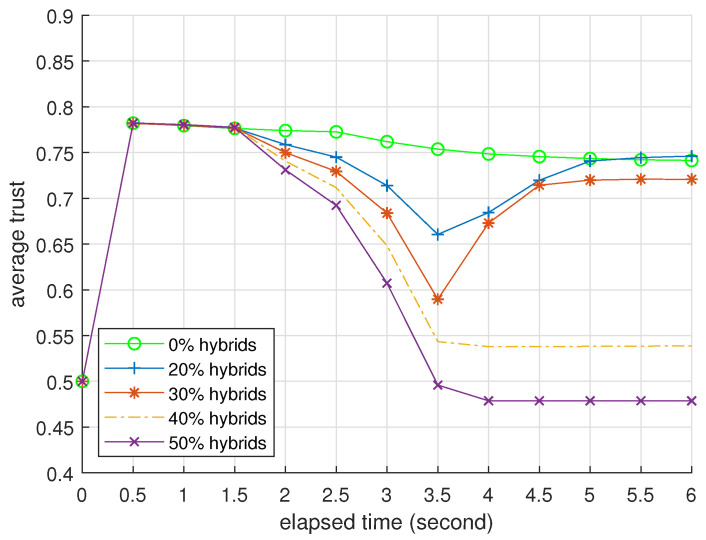
Average trusts of normal devices per 0.5 s, $n_{0}=50$; all attackers are hybrids.

Table 4 and Table 5 present BTM’s performance as an identifier when challenged by foxes and hybrids. We omit the data of misers because they are similar to the data of foxes. Figure 3, Figure 4 and Figure 5 record how the average trusts of normal devices change as time goes by when challenged by foxes, misers, and hybrids. We will interpret these data at the end of this subsection.

In Table 4, the columns of F1 score and accuracy indicate that BTM can thoroughly and correctly distinguish independent BMA attackers from normal devices. This detection ability has the best performance in recall and strengthens with the number of devices, while the proportion of attackers mainly determines its cost of computing resources. In Figure 3, the curve of 0% foxes shows that occasional interaction failure and the side effect of Algorithm 4 cause a deviation in the normal device’s average trust, whose order of magnitude is 0.01. Foxes shift to the attack mode one after another, leading to the decline in the other four curves. The normal device’s average trust is still higher than ζ in the worst case. The main reason probably is the mathematical base for the feedback integration. Moreover, Algorithm 4 lets foxes pay a price of its trust for criticizing other devices, rendering its feedback less persuasive. According to the column of average attacker trust in Table 4, a fox’s trust drops faster than a normal device to let Algorithm 5 check the former earlier. Therefore, the advantage of Algorithm 4 is far greater than its side effect. Algorithm 3 gradually restores the normal device’s trust when the proportion of foxes decreases. Because the accuracy and F1 score are almost in unity, the normal device’s trust can fully recover from BMAs. Moreover, the recovery rates of the four curves are similar. This result corroborates that BTM is robust and resilient against independent BMAs.

In Figure 4, colluding DAs from misers can amplify the side effect of Algorithm 4. Still, the normal device’s average trust is higher than ζ and fully recovers in the worst case. The miser’s trust also drops faster than the normal device. This result corroborates that misers cannot overcome normal devices.

Table 5 indicates the upper limit of BTM’s protection against trust attacks. Hybrids can occupy small networks with a casualty rate of about $\frac{1}{4}$ in half of all devices, but this casualty becomes very heavy when $n_{0}=50$. In Figure 5, the trends of the latter three curves are different from Figure 3 and Figure 4 because BTM cannot guarantee specificity in these cases. When the proportion of hybrids is 30%, the fog node misjudges several normal devices and fully restores the trusts of the remaining devices. Note that the normal device’s average trust also includes misjudged ones.

These data also indicate that normal and malicious devices cannot coexist and support an optimization method. The fog node does not need to interact with the device to examine its status in practice when devices that can guarantee service quality are sufficient; just performing imaginary checks always returns successful results regularly to keep the effect of feedback from devices. It is called token mode and can extra counteract the side effect of Algorithm 4. Table 6 presents BTM’s performance against hybrids in this mode.

### 3.3. Comparison with Existing Research

In this subsection, we compare the performance of BTM with a reliable trust computing mechanism (RTCM) [28] and trust-based service management (TBSM) [16]. In both RTCM and TBSM, the forms of forgetting are conventional and similar to (10), and the global trust estimation is the weighted sum of the direct trust estimation from observation and the indirect trust estimation from feedback. RTCM features the utilization of feedback from multiple sources, where a fog node synthesizes direct trust estimations from devices and computes indirect trust estimations. The device requests indirect trust estimations from the fog node when it needs to compute global trust estimations. TBSM features comprehensive protection against trust attacks. As discussed in the Introduction, existing trust mechanisms have a common issue of dealing with trust attacks. The following simulation illustrates BTM’s advantage of reaching a good trade-off between the protection against BMAs and OOAs.

The setting of this simulation is identical to Table 3 except for the interaction success rate being 1 and ζ=0 to turn off the trust attack detection. There are five devices where devices 0, 1, and 2 are normal, but devices 3 and 4 are attackers. The program records trust estimations per 10 milliseconds, averaged from 2000 trials.

**Figure 6:**
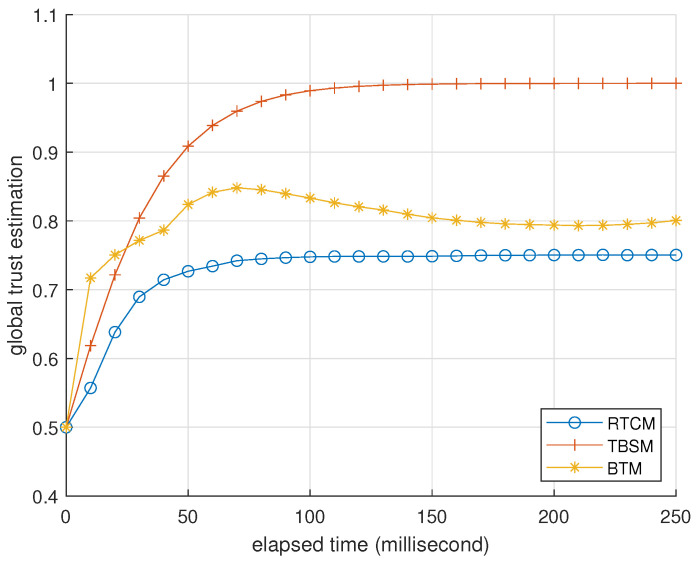
Average global trust estimations of devices 1 and 2 in RTCM, TBSM, and BTM, in the view of device 0, recorded per 10 milliseconds. The forgetting factor is 0.5, and the parameter of indirect trust is 0.5 in RTCM. They are 0.3 and 0.1 in TBSM. ϕ=5 and ζ=0 in BTM.

**Figure 7:**
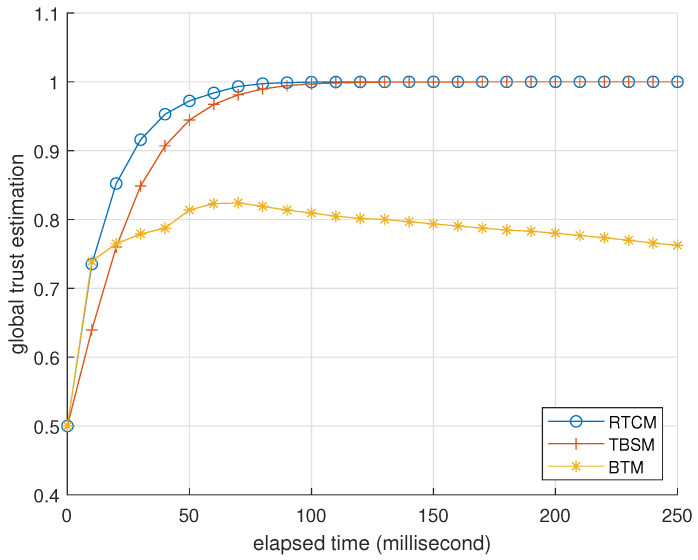
Average global trust estimation of colluding foxes 3 and 4, in the view of device 0, recorded per 10 milliseconds. The parameter setting is identical to Figure 6.

In the first case, devices 3 and 4 are colluding foxes. They launch BMAs against the other three devices. In view of device 0, Figure 6 records the average global trust estimations of devices 1 and 2 in the three trust mechanisms. Figure 7 records the average global trust estimations of attackers 3 and 4. They indicate that attackers can mislead the evaluator into producing trust estimations in more favor of them via BMAs in RTCM, as the fog node does not check the authenticity of feedback from devices. On the contrary, BMAs hardly influences TBSM because it ignores most feedback from the two attackers. TBSM’s idea against BMAs can apply to Algorithm 1: Evaluator *i* calculates $\left(t_{k}^{i}-t_{k}^{j}\right)/t_{k}^{i}$ when it receives ${\mathrm{rep}}_{k}^{j}$. It ignores ${\mathrm{rep}}_{k}^{j}$ if the result transcends 0.5. However, this idea can lead to misjudgment when DAs exist, which happens in the following case. The performance of BTM is between RTCM and TBSM, where BMAs cannot bring attackers extra benefits.

**Figure 8:**
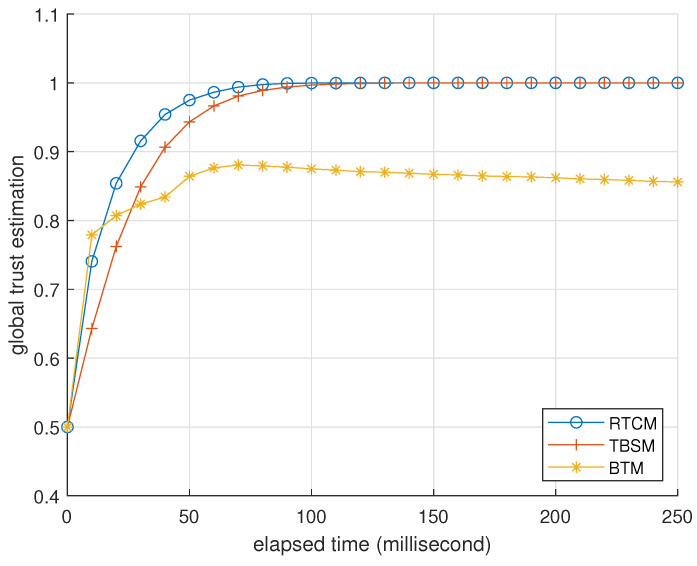
Average global trust estimations of devices 1 and 2, in the view of device 0, recorded per 10 milliseconds. The parameter setting is identical to Figure 6.

**Figure 9:**
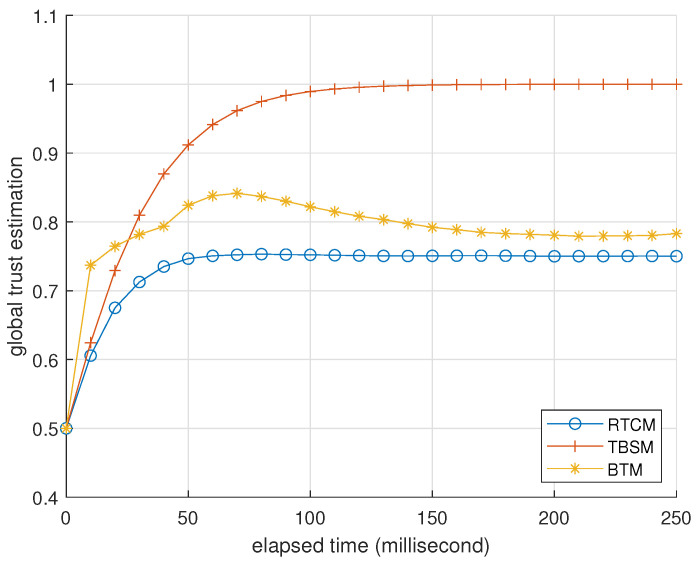
Average global trust estimation of misers 3 and 4, in the view of device 0, recorded per 10 milliseconds. The parameter setting is identical to Figure 6.

In the second case, devices 3 and 4 are misers. They launch DAs against devices 1 and 2 while pretending to be normal in front of device 0. Figure 8 and Figure 9 record the trust estimations corresponding to Figure 6 and Figure 7. They indicate that the performance of RTCM is best to decrease trust estimations of attackers and not influence normal devices. TBSM is unaware of the existence of DAs due to device 0 wrongly regarding feedback from devices 1 and 2 as BMAs. The performance of BTM is between RTCM and TBSM due to the side effect of Algorithm 4, where DAs also cannot bring attackers extra benefits.

## 4. Discussion

The presented simulation results corroborate that BTM’s idea of how to render trust estimations universal and accurate is feasible: assuming that devices frequently communicate with each other and that most of them are normal, the evaluator quantifies a device’s trustworthiness with a strictly probabilistic value, whose updating utilizes direct and external evidence under the guidance of Bayesian statistics. As for the issue of trust attacks, BTM’s feedback integration mechanism based on Jøsang’s belief model features listening to multiple message sources and adopts the principle that it takes two to tango. Therefore, it can mitigate their harm and turn their profit negative when attackers are in the minority. For example, when the proportion of hybrids is 20%, BTM’s performance in accuracy and average attacker trust means that attackers’ trust estimations drop below 0.5 at such a drastic rate that trust attack detection is unnecessary. In environments with high-intensity attacks, the importance of BTM’s trust attack detection mechanism manifests more. These results also confirm that even if simultaneously influenced by inherent uncertainty and trust attacks, BTM can prevent the latter from misleading evaluators into misjudging normal devices. In addition, this paper introduces fog computing to heighten BTM’s scalability. As one motivation for the emergence of fog computing, it helps manage dynamic devices [33] and handle NCAs. For example, when a fog node meets an unacquainted intelligent vehicle, it can request related trust data from a cloud center or nearby fog node using this car’s identifier. There remains room for improving BTM’s security. A valuable research direction is solving the susceptibility to SAs. SA attackers can forge fake identities to spread misinformation whose sources are different. As a result, it can circumvent the second assumption on which BTM depends and successfully achieve trust attacks such as BMAs and BSAs.

## 5. Conclusions

This paper proposes BTM as a lightweight, adaptable, and universal trust mechanism for IoT devices, an enhanced edition of BRS providing more accurate trust estimations and better protection against trust attacks. Based on Bayesian statistics and Jøsang’s belief model, an evaluator updates a device’s reputation vector using direct interaction results and external feedback. A device’s trust estimation comes from its reputation vector. This process can preclude SP attacks and employ fog computing as an optimization technique to address the challenges of managing numerous or dynamic devices. BTM’s forgetting algorithm can automatically set its parameters and ensures that a trust estimation reflects a device’s latest status, retards OOAs, and expedites eliminating influences from trust attacks. BTM’s tango algorithm curbs BMAs with negligible side effects and extra computing by diving the blame of a failing interaction between the two sides during the processing of feedback from different devices. BTM’s trust attack detection based on its trust estimations and hypothesis testing can identify BMAs, BSAs, DAs, and VIE. The simulation results corroborate that BTM can deal with colluding attackers having multiple abilities of BMAs, BSAs, and DAs if most devices are normal.

## Figures and Tables

**Table 1:** Coverage of trust attacks in existing trust mechanisms.

Ref.	OOA	BMA	BSA	DA	SP	VIE	SA	NCA
[8]	✓	✓	✓	-	-	-	-	-
[10]	✓	-	-	-	-	-	-	-
[11]	✓	✓	-	-	-	-	-	-
[13]	✓	✓	-	-	-	-	-	-
[16]	✓	✓	✓	✓	✓	-	-	-
[17]	-	-	-	-	-	-	✓	-
[23]	✓	✓	✓	-	✓	-	-	-
[24]	✓	✓	✓	-	✓	-	-	-
[25]	✓	✓	✓	-	✓	-	-	-

**Table 2:** Notations in BTM.

Notation	Explanation	Section
$t_{j}^{i}$	Device *j*’s trust value in evaluator *i*, derived from ${\mathrm{rep}}_{j}^{i}$.	Section 2.2
${\mathrm{rep}}_{j}^{i}$	Device *j*’s reputation vector, saving data of Bayesian inference. ${\mathrm{rep}}_{j}^{i}=\left(\alpha_{j}^{i},\beta_{j}^{i},r_{j}^{i},s_{j}^{i}\right)$.	
$\alpha_{j}^{i}$ and $\beta_{j}^{i}$	Two hyperparameters of a beta prior distribution.	
$r_{j}^{i}$ and $s_{j}^{i}$	Two parameters saving all evidence in Bayesian inference.	
$o_{j}^{i}$	Evaluator *i*’s opinion about device *j*, defined by Jøsang’s belief model. $o_{j}^{i}=\left(b_{j}^{i},d_{j}^{i},u_{j}^{i}\right)$.	Section 2.3
$b_{j}^{i}$, $d_{j}^{i}$, and $u_{j}^{i}$	Three parameters expressing the extent of belief, disbelief, and uncertainty about device *j*.	
$o_{k}^{i:j}$	Evaluator *i*’s opinion about device *k* after it receives and discounts $o_{k}^{j}$ as feedback.	
$U_{o}$	The opinion set.	
⊗	A binary operation of discounting opinions, defined upon $U_{o}$.	
$U_{r}$	A subset of the reputation vector set, where α and β are constants.	
⊕	A binary operation of merging evidence, defined upon $U_{r}$.	
$g({\mathrm{rep}}_{j}^{i})$	A mapping from $U_{r}$ to $U_{o}$.	
$g^{-1}\left(o_{j}^{i}\right)$	The inverse mapping of *g*.	
$\Delta {\mathrm{rep}}_{j}^{i}=\left(\Delta r_{j}^{i},\Delta s_{j}^{i}\right)$	An increment of ${\mathrm{rep}}_{j}^{i}$, new evidence gathered from recent interactions with devices *j*.	
${\mathrm{evi}}_{k}^{i:j}=\left(m_{k}^{i:j},n_{k}^{i:j}\right)$	All external evidence of device *k* provided by device *j*, ${\mathrm{evi}}_{k}^{i:j}=\sum_{l=1}^{n}\Delta {\mathrm{rep}}_{kl}^{j}$.	
λ	The forgetting factor in the conventional form of forgetting algorithms in current research.	Section 2.4
$q_{j}^{i}$	The evidence queue of device *j*.	
ϕ	The capacity of q.	
$M^{i}$ and $N^{i}$	Evaluator *i* saves external evidence in these two matrices. ${\mathrm{evi}}_{k}^{i:j}=\left(M^{i}\left[j\right]\left[k\right],N^{i}\left[j\right]\left[k\right]\right)$.	
ω	A test statistic of hypothesis testing related to trust attack detection.	Section 2.5
ζ	Evaluator *i* does not check whether device *j* is a trust attacker if $t_{j}^{i}>\zeta$.	
$\gamma_{1}$	A significance level used to identify restricted BMAs, BSAs, or VIE, as well as DAs.	
$\gamma_{2}$	A very tiny significance level used to identify reckless BMAs, BSAs, or VIE.	
η	Evaluator *i* judges device *j* as suspicious if $\omega <\gamma_{1}$ happens more than η in a check.	

**Table 3:** Simulation and algorithm parameters.

Parameter	Value
interaction success rate	0.8
$n_{0}$, initial device number	10, 20, and 50
device sleep after sending a request	1 millisecond
max request sending count for devices	20 $n_{0}$
*n*, active device number	variable, from $n_{0}$ to 0
periodical fog node sleep	automatically adjusted variable
request sending count as latency for foxes	variable for each fox, $\left[0,5n_{0}\right]$
latency for misers and hybrids	$5n_{0}$
ϕ	5
ζ	0.6
$\gamma_{1}$	0.03125
$\gamma_{2}$	$2\times 10^{-6}$
η	variable, $max\left(3,\left\lfloor 0.2n\right\rfloor \right)$

**Table 4:** Data of the fox, round off to five decimal places.

Device Number	Percentage	Precision	Recall	Specificity	Accuracy	F1 Score	Average Deviation	Average Attacker Trust	Check Count
10	0%	-	-	0.99950	0.99950	-	0.05149	-	9.02900
	20%	0.98138	0.99225	0.99300	0.99285	0.98679	0.05665	0.54100	25.66100
	30%	0.96588	0.99017	0.97893	0.98230	0.97787	0.06371	0.52258	42.11600
	40%	0.91389	0.98387	0.91396	0.94192	0.94759	0.08621	0.50595	63.81491
	50%	0.83569	0.96110	0.75430	0.85770	0.89402	0.13325	0.49487	85.20300
20	0%	-	-	1.00000	1.00000	-	0.05087	-	5.54900
	20%	0.99980	1.00000	0.99994	0.99995	0.99990	0.04886	0.56118	20.31600
	30%	0.99936	1.00000	0.99968	0.99978	0.99968	0.05016	0.54927	31.37000
	40%	0.99383	0.99994	0.99533	0.99718	0.99688	0.05444	0.53335	49.59000
	50%	0.97545	0.99985	0.97195	0.98590	0.98750	0.06530	0.51789	76.28400
50	0%	-	-	1.00000	1.00000	-	0.05908	-	3.57000
	20%	1.00000	1.00000	1.00000	1.00000	1.00000	0.05697	0.55582	33.25700
	30%	0.99991	1.00000	0.99996	0.99997	0.99995	0.05674	0.54782	48.17000
	40%	0.99868	1.00000	0.99907	0.99944	0.99934	0.05037	0.53483	69.76400
	50%	0.97508	1.00000	0.97180	0.98590	0.98738	0.05788	0.52091	109.97900

**Table 5:** Data of the hybrid, round off to five decimal places.

Device Number	Percentage	Precision	Recall	Specificity	Accuracy	F1 Score	Average Deviation	Average Attacker Trust	Check Count
10	0%	-	-	0.99950	0.99950	-	0.05149	-	9.02900
	20%	0.98975	1.00000	0.99613	0.99690	0.99485	0.05452	0.44899	16.34600
	30%	0.95886	1.00000	0.97479	0.98235	0.97900	0.06400	0.45518	27.00000
	40%	0.76964	0.97150	0.74075	0.83305	0.85887	0.13228	0.47813	51.55800
	50%	0.16931	0.24250	0.04710	0.14480	0.19940	0.31830	0.81486	48.39600
20	0%	-	-	1.00000	1.00000	-	0.05087	-	5.54900
	20%	0.99960	1.00000	0.99988	0.99990	0.99980	0.04852	0.46585	17.23200
	30%	0.98064	1.00000	0.98993	0.99295	0.99023	0.05188	0.48106	35.51700
	40%	0.71191	0.99969	0.68292	0.80963	0.83161	0.13518	0.48967	87.55778
	50%	0.18022	0.25575	0.00060	0.12818	0.21144	0.31723	0.84635	70.64000
50	0%	-	-	1.00000	1.00000	-	0.05908	-	3.57000
	20%	0.99959	1.00000	0.99989	0.99991	0.99979	0.05508	0.47747	24.88400
	30%	0.70225	1.00000	0.80037	0.86026	0.82508	0.08603	0.48928	114.31800
	40%	0.40842	1.00000	0.03390	0.42034	0.57997	0.28827	0.48234	149.01100
	50%	0.47261	0.90072	0.00000	0.45036	0.61994	0.32128	0.57290	141.57200

**Table 6:** Data of the hybrid in token mode, round off to five decimal places.

Device Number	Percentage	Precision	Recall	Specificity	Accuracy	F1 Score	Average Deviation	Average Attacker Trust	Check Count
10	0%	-	-	1.00000	1.00000	-	0.03112	-	0.76900
	20%	1.00000	1.00000	1.00000	1.00000	1.00000	0.03509	0.46025	4.37300
	30%	1.00000	1.00000	1.00000	1.00000	1.00000	0.03791	0.46532	7.63200
	40%	0.97456	0.99500	0.97733	0.98440	0.98467	0.04884	0.48325	26.13600
	50%	0.03004	0.03400	0.01630	0.02515	0.03190	0.31156	0.98334	29.45200
20	0%	-	-	1.00000	1.00000	-	0.03032	-	0.95800
	20%	1.00000	1.00000	1.00000	1.00000	1.00000	0.02860	0.48138	7.95900
	30%	0.99979	1.00000	0.99989	0.99993	0.99989	0.02874	0.49852	15.35900
	40%	0.88543	1.00000	0.89929	0.93958	0.93923	0.05402	0.50189	61.66700
	50%	0.08593	0.10685	0.00050	0.05367	0.09525	0.30542	0.95278	48.56400
50	0%	-	-	1.00000	1.00000	-	0.04580	-	0.88800
	20%	1.00000	1.00000	1.00000	1.00000	1.00000	0.03991	0.48726	16.99500
	30%	0.85949	1.00000	0.92164	0.94515	0.92444	0.05045	0.50205	68.19900
	40%	0.44513	1.00000	0.16505	0.49903	0.61604	0.25174	0.48640	129.41300
	50%	0.43451	0.77068	0.00000	0.38534	0.55571	0.31680	0.65250	118.11700

## Data Availability

The source code of the simulation program and the raw data are available from the corresponding author upon request.

## References

[1] Balaji S., Nathani K., Santhakumar R. (2019). IoT technology, applications and challenges: A contemporary survey. Wirel. Pers. Commun..

[2] Gu L., Wang J., Sun B. (2014). Trust management mechanism for Internet of Things. China Commun..

[3] Gambetta D. (2000). Can we trust trust. Trust: Making and Breaking Cooperative Relations.

[4] Hassija V., Chamola V., Saxena V., Jain D., Goyal P., Sikdar B. (2019). A survey on IoT security: Application areas, security threats, and solution architectures. IEEE Access.

[5] Altaf A., Abbas H., Iqbal F., Derhab A. (2019). Trust models of Internet of Smart Things: A survey, open issues, and future directions. J. Netw. Comput. Appl..

[6] Atzori L., Iera A., Morabito G., Nitti M. (2012). The social Internet of Things (SIoT)—When social networks meet the Internet of Things: Concept, architecture and network characterization. Comput. Netw..

[7] Shafer G. (1976). A Mathematical Theory of Evidence.

[8] Ganeriwal S., Balzano L.K., Srivastava M.B. (2008). Reputation-based framework for high integrity sensor networks. ACM Trans. Sens. Netw..

[9] Raya M., Papadimitratos P., Gligor V.D., Hubaux J.P. (2008). On data-centric trust establishment in ephemeral ad hoc networks. Proceedings of the IEEE INFOCOM 2008-the 27th Conference on Computer Communications.

[10] Wei Z., Tang H., Yu F.R., Wang M., Mason P. (2014). Security enhancements for mobile ad hoc networks with trust management using uncertain reasoning. IEEE Trans. Veh. Technol..

[11] Li W., Song H. (2015). ART: An attack-resistant trust management scheme for securing vehicular ad hoc networks. IEEE Trans. Intell. Transp. Syst..

[12] Meng W., Choo K.K.R., Furnell S., Vasilakos A.V., Probst C.W. (2018). Towards Bayesian-based trust management for insider attacks in healthcare software-defined networks. IEEE Trans. Netw. Serv. Manag..

[13] Anwar R.W., Zainal A., Outay F., Yasar A., Iqbal S. (2019). BTEM: Belief based trust evaluation mechanism for wireless sensor networks. Future Gener. Comput. Syst..

[14] Soleymani S.A., Abdullah A.H., Zareei M., Anisi M.H., Vargas-Rosales C., Khan M.K., Goudarzi S. (2017). A secure trust model based on fuzzy logic in vehicular ad hoc networks with fog computing. IEEE Access.

[15] Jiang J., Han G., Zhu C., Chan S., Rodrigues J.J. (2017). A trust cloud model for underwater wireless sensor networks. IEEE Commun. Mag..

[16] Chen R., Bao F., Guo J. (2015). Trust-based service management for social Internet of Things systems. IEEE Trans. Dependable Secur. Comput..

[17] Awan K.A., Din I.U., Almogren A., Guizani M., Khan S. (2020). StabTrust: A stable and centralized trust-based clustering mechanism for IoT enabled vehicular ad-hoc networks. IEEE Access.

[18] Dedeoglu V., Jurdak R., Putra G.D., Dorri A., Kanhere S.S. A trust architecture for blockchain in IoT. Proceedings of the 16th EAI International Conference on Mobile and Ubiquitous Systems: Computing, Networking and Services.

[19] Shala B., Trick U., Lehmann A., Ghita B., Shiaeles S. (2020). Blockchain and trust for secure, end-user-based and decentralized IoT service provision. IEEE Access.

[20] Malik S., Dedeoglu V., Kanhere S.S., Jurdak R. (2019). Trustchain: Trust management in blockchain and IoT supported supply chains. Proceedings of the 2019 IEEE International Conference on Blockchain.

[21] Ullah F., Pun C.M., Kaiwartya O., Sadiq A.S., Lloret J., Ali M. (2023). HIDE-Healthcare IoT data trust managEment: Attribute centric intelligent privacy approach. Future Gener. Comput. Syst..

[22] Haseeb K., Rehman A., Saba T., Bahaj S.A., Wang H., Song H. (2023). Efficient and trusted autonomous vehicle routing protocol for 6G networks with computational intelligence. ISA Trans..

[23] Ogundoyin S.O., Kamil I.A. (2021). A trust management system for fog computing services. Internet Things.

[24] Junejo A.K., Komninos N., Sathiyanarayanan M., Chowdhry B.S. (2019). Trustee: A trust management system for fog-enabled cyber physical systems. IEEE Trans. Emerg. Top. Comput..

[25] Alemneh E., Senouci S.M., Brunet P., Tegegne T. (2020). A two-way trust management system for fog computing. Future Gener. Comput. Syst..

[26] Chiang M., Zhang T. (2016). Fog and IoT: An overview of research opportunities. IEEE Internet Things J..

[27] Wang T., Zhang G., Bhuiyan M.Z.A., Liu A., Jia W., Xie M. (2020). A novel trust mechanism based on fog computing in sensor–cloud system. Future Gener. Comput. Syst..

[28] Liang J., Zhang M., Leung V.C. (2020). A reliable trust computing mechanism based on multisource feedback and fog computing in social sensor cloud. IEEE Internet Things J..

[29] Zhang G., Wang T., Wang G., Liu A., Jia W. (2021). Detection of hidden data attacks combined fog computing and trust evaluation method in sensor-cloud system. Concurr. Comput. Pract. Exp..

[30] Hussain Y., Zhiqiu H., Akbar M.A., Alsanad A., Alsanad A.A.A., Nawaz A., Khan I.A., Khan Z.U. (2020). Context-aware trust and reputation model for fog-based IoT. IEEE Access.

[31] Rathee G., Sandhu R., Saini H., Sivaram M., Dhasarathan V. (2020). A trust computed framework for IoT devices and fog computing environment. Wirel. Netw..

[32] Fang W., Zhang W., Chen W., Liu Y., Tang C. (2020). TMSRS: Trust management-based secure routing scheme in industrial wireless sensor network with fog computing. Wirel. Netw..

[33] Yannuzzi M., Milito R., Serral-Gracià R., Montero D., Nemirovsky M. (2014). Key ingredients in an IoT recipe: Fog computing, cloud computing, and more fog computing. Proceedings of the 2014 IEEE 19th International Workshop on Computer Aided Modeling and Design of Communication Links and Networks.

[34] Josang A., Ismail R. The beta reputation system. Proceedings of the 15th Bled Electronic Commerce Conference.

[35] Wang H., Bah M.J., Hammad M. (2019). Progress in outlier detection techniques: A survey. IEEE Access.

[36] Breunig M.M., Kriegel H.P., Ng R.T., Sander J. LOF: Identifying density-based local outliers. Proceedings of the 2000 ACM SIGMOD International Conference on Management of Data.

